# Outcomes of Castor single-branched stent graft combined with in situ fenestration left subclavian artery in aortic arch disease: a single-center experience

**DOI:** 10.1186/s42155-025-00637-9

**Published:** 2026-01-24

**Authors:** Fanyun Liu, Jianming Sun, Yikuan Chen, Xiaotong Qi, Hailong Luo

**Affiliations:** Department of Vascular Surgery, The Second Affiliated Hospital of Chongqing Medical University, No 76, Linjiang Road, Yuzhong District, Chongqing, China

**Keywords:** Aortic arch, Aortic arch branches, In situ laser fenestration, Thoracic endovascular aortic repair, Castor single-branched stent-graft

## Abstract

**Background:**

To evaluate outcomes of Castor single-branched stent graft combined with in situ fenestration of the left subclavian artery for aortic arch disease.

**Methods:**

A retrospective analysis of 30 patients undergoing TEVAR with Castor stent that was first implanted with the branch in the left common carotid artery (LCCA) followed by in situ fenestration of the left subclavian (LSA) between March 2022 and March 2024 was conducted. Perioperative and follow-up data were collected and analyzed.

**Results:**

The study retrospective analysis of 30 patients comprised 16 (53.3%) with acute type B aortic dissections, 6 (20%) with thoracic aortic aneurysms, 2 (6.7%) with intramural hematoma, and 6 (20%) with penetrating aortic ulceration. The technical success rate was 96.7% (29/30). One patient required carotid-axillary bypass due to subclavian artery lateral wall opening. There were no cases of mortality, stroke, upper limb ischemia, paraplegia, or stent graft-induced new entry within the 30-day follow-up period. Median hospitalization was 11 (IQR, 9–14) days, with a median follow-up of 12 (IQR, 8–19) months. One patient (3.3%) suffered a fall-related cerebral hemorrhage unrelated to the procedure. Another (3.3%) developed left upper limb ischemia due to stent angulation, corrected with a cover stent. Subclavian artery patency was 96.6% (28/29), and carotid artery patency was 100%. No deaths, endoleaks, or stent migrations occurred.

**Conclusion:**

The Castor stent combined with in situ fenestration is a feasible, effective, and safe strategy for aortic arch disease repair, especially in providing alternative approaches for aortic diseases that require reconstruction in both branches.

## Background

Aortic arch diseases involving branches present significant challenges in vascular and cardiac surgery due to the complexity of the procedures, high perioperative complications, and mortality rates [1]. Traditional surgical methods for aortic arch involvement, such as artificial vascular replacement under cardiopulmonary bypass, hybrid procedures, and debranching techniques, have been associated with neurological complications and surgical trauma [2–4] and mortality and stroke rates as high as 15% [5]. However, with advancements in surgical techniques and perioperative care, the 30-day mortality rate for patients undergoing thoracic aortic repair was 10.7% [6].

Thoracic endovascular aortic repair (TEVAR) has revolutionized the management of thoracic aortic disease, significantly reducing perioperative morbidity and mortality compared to conventional open surgical repair [7, 8]. However, the absence of an adequate proximal landing zone can make standard endovascular repair of the thoracic aorta inappropriate [9]. Previous studies have indicated that TEVAR typically requires a proximal landing zone of 1.5–2.0 cm to ensure safe stent sealing [10]. Aortic arch disease presents additional complexity, as short proximal necks and limited landing zones remain challenging. To achieve an adequate proximal landing zone, covering the aortic arch branches is often necessary, but this approach increases the risk of neurological complications by potentially decreasing cerebral blood flow [11, 12]. While various techniques for reconstructing multiple branches of the aortic arch have demonstrated good clinical outcomes [1, 13, 14], a standard surgical plan has yet to be established. Currently, the ESVS/EACTS expert consensus provides the only recommendations for the treatment of aortic arch diseases, specifically addressing total endovascular aortic arch repair for aortic diseases [15]. The above-mentioned techniques for reconstructing branched vessels are highly challenging, with a high incidence of complications, making them difficult to widely adopt. Therefore, there is a need in clinical practice to explore safer and simpler methods of reconstruction.

The Castor stent (MicroPort Medical, Shanghai, China), a novel single-branched stent graft designed to preserve single-branch vessels in the aortic arch, has demonstrated safety and effectiveness [16]. Currently, there are limited reports on the use of Castor single-branched stent graft combined with in situ fenestration for treating aortic arch diseases [1]. This study found that the Castor stent, when used in conjunction with in situ fenestration, yields favorable clinical outcomes, particularly in reconstructing left common carotid artery. This study aims to evaluate the outcomes of endovascular repair for thoracic aortic lesions involving Ishimaru zone 1 [17] using the Castor approach combined with the in situ fenestration technique.

## Materials and methods

### Patients

The institutional review board approved the retrospective study with a waiver of informed consent. This single-center, retrospective study focused on aortic arch diseases with simultaneous reconstruction of the left common carotid artery and left subclavian artery. The study reviewed consecutive 30 patients with aortic arch disease who underwent TEVAR using a Castor single-branched stent graft that the single branch was placed in the left common carotid artery with left subciavian in situ fenestration between March 2022 and March 2024 in our center. The inclusion criteria were as follows: (1) age 18 to 80 years, (2) aortic arch disease, (3) reconstruction of a double arch branch. The exclusion criteria were as follows: (1) lack of an appropriate access route for the stent graft, (2) aortic caliber that did not match the Castor single-branched stent graft, (3) those who cannot tolerate general anesthesia by TEVAR, (4) previous history of TEVAR. Key indications for this approaches was landing zone located in Ishimaru zones 1. Patient demographics, operative and postoperative details, follow-up, and outcomes were retrospectively reviewed.

### Pre-operative evaluation

**Fig. 1:**
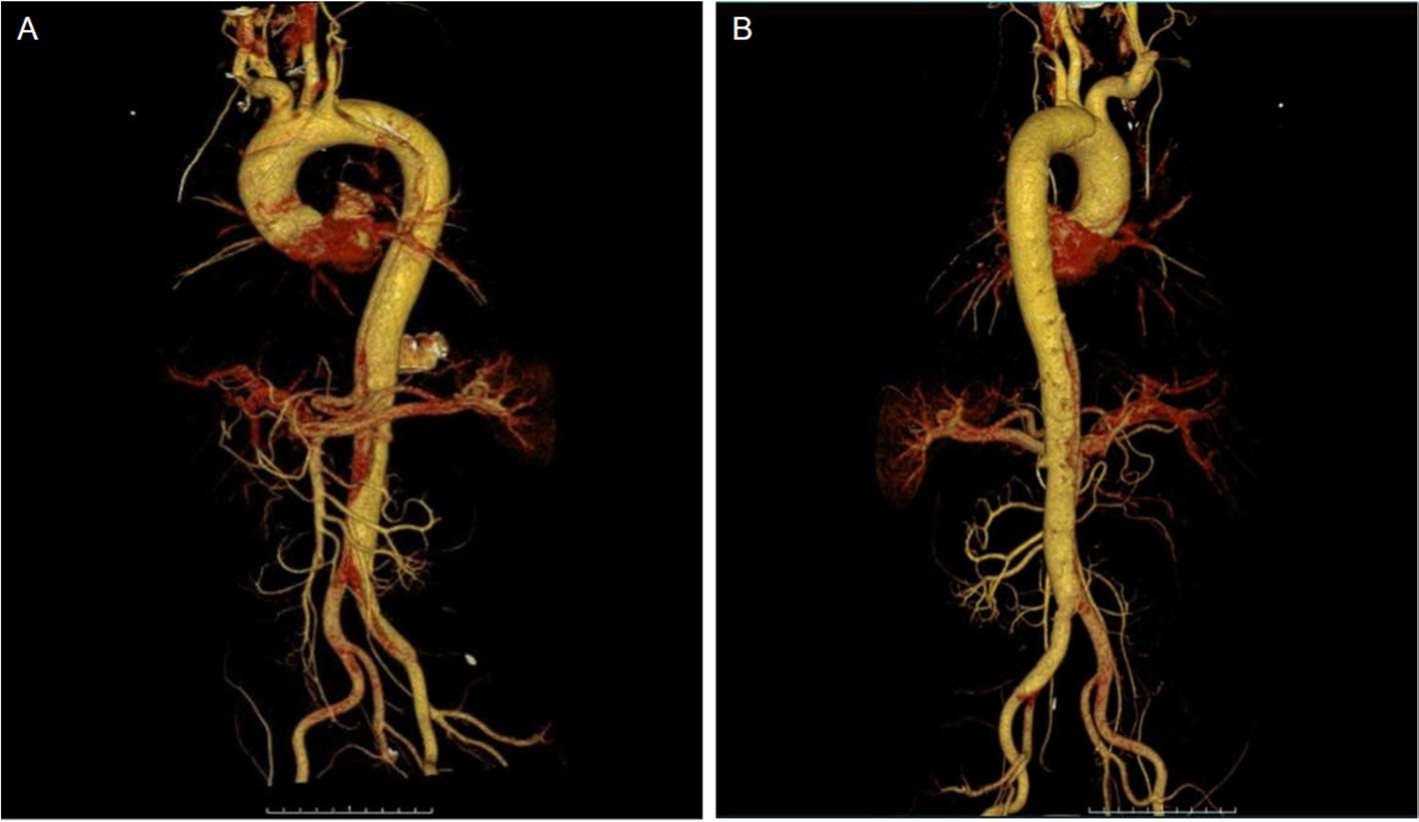
**A** and **B** Preoperative computed tomography angiography with 3-dimensional reconstruction of aortic dissection involved the zone 2 and reconstruction of the left common carotid artery was required

Preoperative thoracic and abdominal aortic computed tomography angiography (CTA) was performed in all patients (Fig. 1). The CTA provided measurements of the distance to the branch of the aortic arch, branch vessel diameter, aortic arch diameter, landing zone diameter, and descending aorta diameter.

### Definitions

#### Technical success

Successful Castor stent deployment, patency of the left subclavian artery fenestration (confirmed by intraoperative angiography), absence of type I/III endoleaks, and no intraprocedural major adverse events (stroke, aortic rupture, death).

### Endovascular procedure

**Fig. 2:**
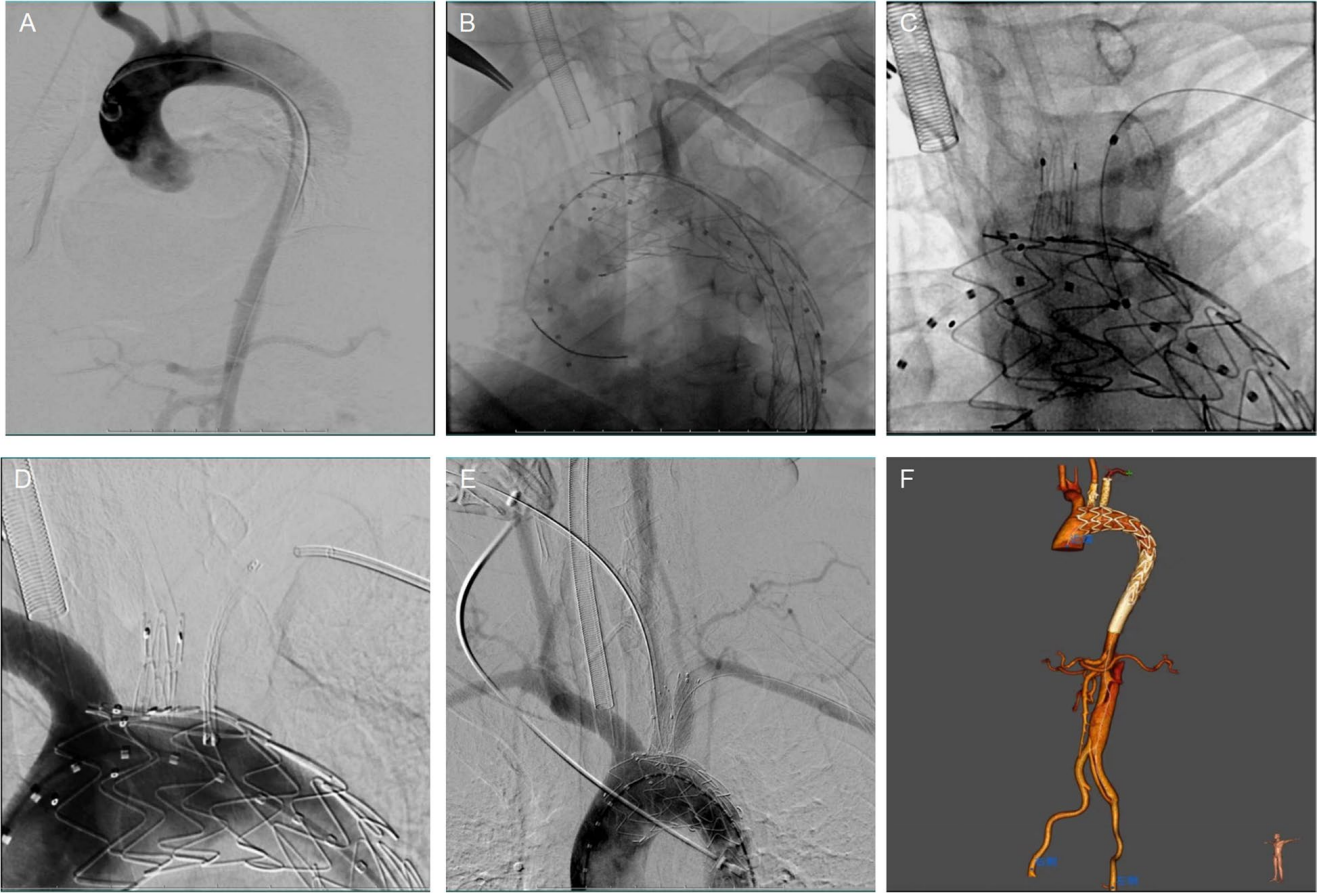
**A** Preoperative aortography of aorta. **B** Determine the Angle and position of the window. **C** In situ laser fenestration of LSA and balloon dilatation performed to enlarge the fenestration. **D** LSA stent placement. **E** Aortography showed that the branch arteries were patent. **F** Postoperative CTA showed that the branch stent was patent, and the false lumen of the thoracic aorta was completely thrombosis

All patients received general anesthesia and complete heparinization (100 units/kg) during the procedure. Ultrasound-guided percutaneous micropuncture access was achieved through the common femoral artery (CFA), and ProGlide (Abbott Vascular, CA, USA) was used. The left common carotid artery (LCCA) and left brachial artery (LBA) were exposed surgically, and a 6 F vascular sheath was inserted into both the LCCA and LBA. Ascending aorta angiography was performed initially to assess the location, nature, and extent of the lesion (Fig. 2A). Bare stent placement considered based on distal lumen diameter to expand the true lumen and reduce distal stent graft-induced new entry in case of dissection. The Castor release technique has been previously described [16]. Briefly, a catheter access was established between the LCCA and the CFA on the operating side. An appropriate Castor single-branched stent graft was selected, and the branch guide wire was advanced from CFA guide catheter to LCCA. The Castor stent graft was introduced into the target position in the aorta along a super stiff guidewire, and the branch stent guide wire was guided through the connecting catheter while the traction wire of the branch section was simultaneously withdrawn from the sheath in the LCCA. Once the delivery system reached the beginning of the descending aorta, the stent graft trunk was positioned in the aortic arch. After ensuring that there was no wrapping between the guide wires of the main and branch stents, the tip of the proximal stent was advanced to the predetermined landing zone (behind the brachiocephalic trunk artery opening), and the main stent and branch stent were released sequentially. Angiography was then performed to confirm the stent positions, patency of the branch stents, and the presence of endoleak.

In situ fenestration using a laser technique has been described in previous articles [18]. After deploying the endograft, angiography was performed to identify optimal fenestration site (Fig. 2B). A 4 × 40-mm Mustang balloon catheter (Boston Scientific, MA) was prepared by inserting an 810-nm optical fiber through its inner lumen (a coaxial setup that protects the fiber and ensures precise alignment); this balloon-fiber assembly was then gently advanced through a pre-placed vascular sheath (8F/55 cm) and navigated under continuous fluoroscopy until the balloon’s distal end (and the fiber tip) reached the pre-identified fenestration site on the endograft wall. Once stabilized against the endograft, laser energy was applied at 14 to 18 W for 3 s via the optical fiber to create a controlled fenestration in the endograft’s fabric. Once the in situ fenestration was created, a V-18 wire was advanced into the ascending aorta through the central lumen. Balloon dilation was performed to enlarge the fenestration, using a 0.035-inch stiff guidewire. The fenestration was sequentially dilated with 4 mm, 6 mm, and 8 mm diameter balloons as needed (Fig. 2C). Finally, a bridging stent [Fluency (CR Bard, Murray Hill, NJ, USA), Lifetream (WL Gore, Flagstaff, AZ, USA)] was deployed within the fenestration (Fig. 2D). An additional balloon was deployed to relieve the stenosis if there was apparent residual stenosis after the initial bridging stent placement. Withdrew delivery system and confirmed patency of the stent, branch, and fenestrated stent via ascending aortography (Fig. 2E).

Subsequently, the catheter, guidewire, and vascular sheath were carefully removed, and meticulous hemostasis measures were taken. The left common carotid artery and left brachial artery were sutured, and no bleeding was observed. The incision was closed layer by layer. Patients were prescribed aspirin (100 mg per day) following TEVAR.

### Follow-up

After the intervention, clinical outcomes, operating time, technical success, any type of endoleak, and related complications were recorded. Routine follow-up included CTA scans at 1, 6, and 12 months, and annually thereafter.

### Statistical analysis

Patient demographics, the surgical data, clinical outcomes, and follow-up data were analyzed using SPSS software, Version 20 (SPSS Inc., Chicago, IL, USA). Normally distributed data are presented as mean values ± standard deviation (SD), non-normally distributed data as median (interquartile range, IQR), and categorical variables as number (percentage, %). Kaplan–Meier analysis was used to generate estimates for the patency rate for the subclavian artery branch and freedom from complications and reinterventions.

## Results

### Demographics

Thirty consecutive patients underwent underwent TEVAR using the Castor stent combined with in situ fenestration. Details of patients are listed in Table 1.

**Table 1:** Baseline characteristics of 30 patients

Variable	Value
Age (years)	60 ± 13.6
Male gender, *n* (%)	26 (86.7%)
Body mass index (kg/m^2^)	22.6 ± 2.8
Comorbidity	
Hypertension, *n* (%)	22 (73.3%)
Diabetes mellitus, *n* (%)	2 (6.7%)
Chronic obstructive pulmonary disease, *n* (%)	6 (20%)
Coronary artery disease, *n* (%)	5 (16.7%)
Prior stroke, *n* (%)	0
Chronic kidney disease, *n* (%)	2 (6.7%)
Limb ischemia, *n* (%)	0
Previous aortic repair, *n* (%)	0
Tobacco abuse, *n* (%)	15 (50%)
Disease type	
Acute type B Aortic dissection, *n* (%)	16 (53.3%)
Intramural haematoma, *n* (%)	2 (6.7%)
Penetrating aortic ulceration, *n* (%)	6 (20%)
Thoracic aortic aneurysms n(%)	6 (20%)
Type of arch	
Type I	9 (30%)
Type II	13 (43.3%)
Type III	8 (26.7%)

### Perioperative results and 30-day outcomes

Detailed surgical data are presented in Table 2. The mean procedural time was 120.7 ± 33.6 min. All procedures were successful except for one technical failure: the subclavian artery was opened at the lateral wall of the aortic arch, and the window could not be successfully created, necessitating a carotid-axillary artery bypass. Restrictive bare stents (26.7%) were used to address severe true lumen stenosis of the aortic dissection and protect the intima at the distal end of the stent graft. No endoleaks were observed during the operation. The median hospitalization time was 11 (IQR, 9–14) days. Perioperative mortality was zero, and no serious complications such as stroke, acute myocardial infarction, renal failure, or left arm ischemia occurred. Aortic CTA after operation showing the branch stent was patent (Fig. 2F).

**Table 2:** The surgical data

Variable	Value
Technical success	96.7%
Operation time, min, mean ± SD	120.7 ± 33.6
Proximal landing zone diameter, mm, mean ± SD	33.8 ± 3.4
Distal landing zone diameter, mm, mean ± SD	**27.8** ± **3.4**
Hospital stay, days, median (IQR)	11 (IQR, 9–14)
Intraoperative fluoroscopy time, min, mean ± SD	73.7 ± 6
Contrast material, ml, mean ± SD	161.5 ± 9.7
LSA diameter, mm, mean ± SD	10.2 ± 1.5
LCCA diameter, mm, mean ± SD	10 ± 1.3
Bridging stents	
Fluency	18 (60%)
Lifestream	11 (36.7%)
Endoleak	
Type Ia	0
Type Ib	0
Type Ⅲ	0
Restrictive bare stent	8 (26.7%)
Laser fenestrations	96.7% (29/30)
Peri-operative	
Stroke	0
Mortality	0
Acute myocardial infarction	0
Renal failure	0
Left arm ischemia	0
Paraplegia	0

*LCCA* left common carotid artery, *LSA* left subclavian artery

The 30-day outcomes after TEVAR are listed in Table 3. There were no cases of mortality, stroke upper limb ischemia, paraplegia, or stent graft-induced new entry within the 30-day follow-up period.

**Table 3:** Thirty-day outcomes after TEVAR

Variable	Value
30-day mortality	0
30-day aortic-related mortality	0
Stroke	1 (3.3%)
Ischemic symptoms of the left arm	0
Paraplegia	0
P-SINE	0
D-SINE	0

*D-SINE* distal stent graft-induced new entry, *P-SINE* proximal stent graft-induced new entry

### Follow up outcomes

All 30 patients were closely followed according to the protocol, with no patients lost to follow-up. The follow-up outcomes are presented in Table 4. The median follow-up period was 12 (IQR, 8–19) months (range 3–25 months). Another patient (3.3%) suffered a cerebral hemorrhage due to a fall 1 month after the procedure, which required tracheotomy and life support. Therefore, the follow-up complication rate was 3.3%. These complications were not related to the stent.

**Table 4:** Follow-up data

Variable	Value
Follow-up time (months)	12 (IQR 8–19)
Follow-up death, *n* (%)	0
Paraplegia (*n*, %)	0
Encephalorrhagia	1 (3.3%)
Stroke	0
Ischemic symptoms of the left upper limb (*n*, %)	1 (3.3%)
P-SINE (*n*, %)	0
D-SINE (*n*, %)	0
Endoleak (*n*, %)	0
Migration of stents (*n*, %)	0
Thoracic aorta false lumen thrombosis (*n*, %)	16 (100%)
Abdominal aorta distal false lumen thrombosis (*n*, %)	4 (25%)
Patency of subclavian artery branch stent (*n*, %)	28 (96.6%)
Patency of left carotid artery branch stent (*n*, %)	30 (100%)
Reintervention, *n* (%)	1 (3.3%)

*D-SINE* distal stent graft-induced new entry, *P-SINE* proximal stent graft-induced new entry

**Fig. 3:**
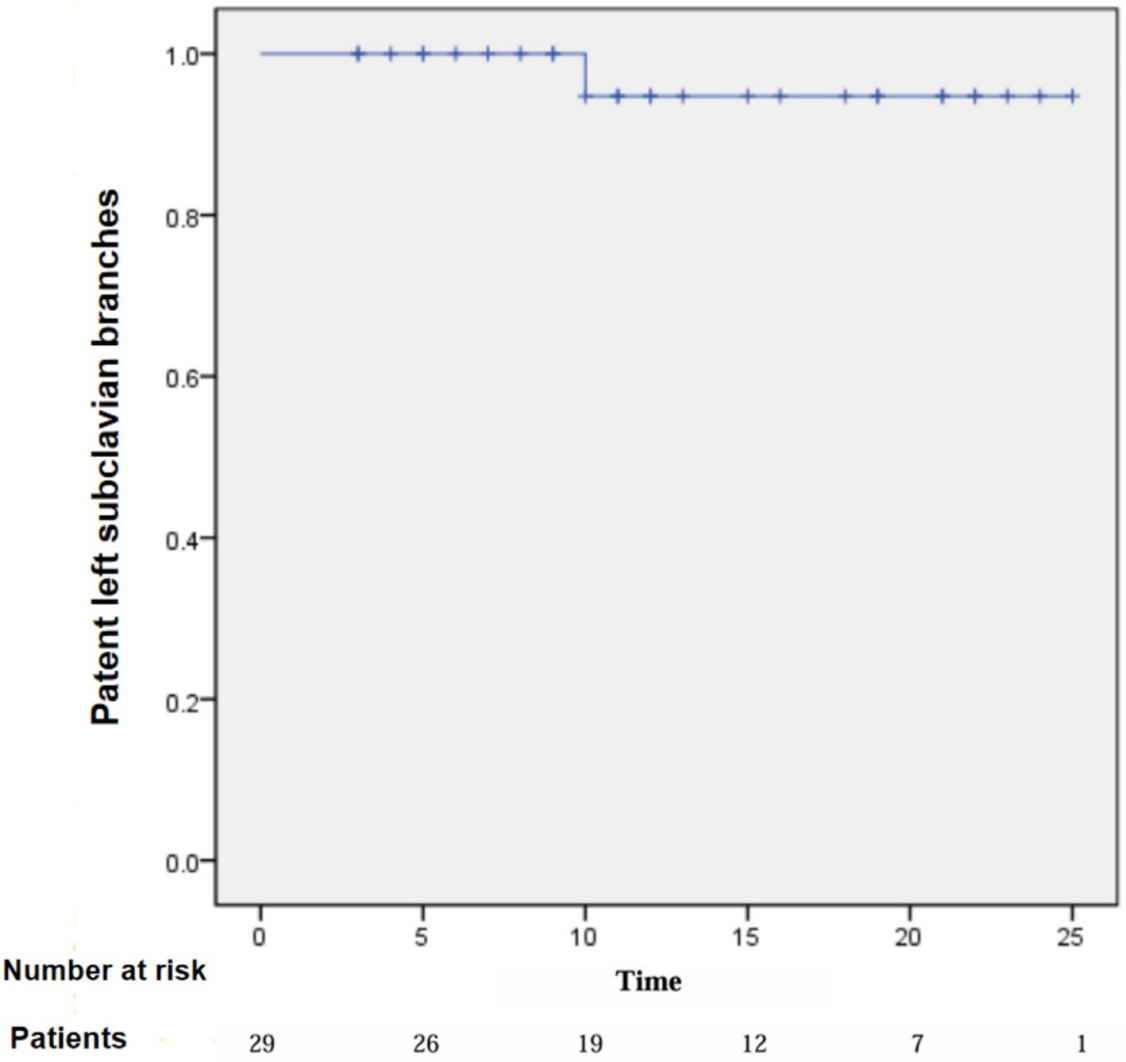
Cumulative Kaplan–Meier estimates of patency rate of the branch section in 29 patients. One patient had stent occlusion within 10 months days after operation

One patient (3.3%) developed left upper extremity ischemia 10 months postoperatively secondary to LSA stent (Fluency) compression with resultant flow obstruction. A corrective stent placement with Lifestream restored patency. The rate of patent left subclavian branches was 96.6% during follow-up (Fig. 3). There were no occlusions observed in the left carotid artery branch stent.

All 16 aortic dissections patients exhibited complete thrombosis of the false lumen in the distal arch and proximal descending aorta, and 4 patients (25%) showed complete thrombosis of the false lumen in the abdominal aorta according to the CTA examination. During the study period, there were no occurrences of death, paraplegia, stent graft-induced new entry, endoleak, or stent migration.

## Discussion

For aortic arch disease, particularly in zone 0, open surgery currently remains the first-line treatment with severe complications, 30-day morbidity and mortality rates of 30% to 50% and 10% to 20%, respectively [6]. Currently, total endovascular treatment of aortic arch diseases is becoming increasingly popular due to its low complication rate [19]. Endovascular repair techniques include branched stent grafts, chimneys, and fenestrations. Despite the experience and skill of the operators, the technical difficulties of side branch catheterization, exacerbated by the need for surgical revascularization of the LSA, create an inherently high risk of cerebral embolism and other adverse outcomes. The Castor stent was developed as a novel single-branched stent graft to preserve single-branched vessels in the arch and has been proven to be safe and effective [16]. However, this approach primarily focuses on left subclavian artery (LSA) reconstruction and fails to address lesions located in the Z2 zone. In contrast, the chimney technique offers advantages such as straightforward operability, no requirement for custom-made devices, and the ability to reconstruct all three aortic arch branches. Its most significant limitation, however, is the high risk of type Ia endoleak.

Previous research reported endoleak rates ranging from 0 to 57% [20]. As a minimally invasive alternative, it represents an urgent option and has not been used as a routine treatment modality. Fenestration technology can be divided into in vitro and in situ fenestration. The advantages of this technology can reduce the occurrence of Type I endoleaks. In vitro fenestration is a modification of traditional aortic grafts based on the anatomical morphology of the aortic arch. Upon release, the fenestration of the graft is aligned with the opening of the branch vessel, thereby preserving the supra-arch branch vessels. Studies on extracorporeal double fenestrations or triple fenestrations have shown that the success rate of extracorporeal multiple fenestrations is approximately 90% and perioperative mortality can reach up to 10% [21]. In situ fenestration is a technique in which the traditional thoracic aortic graft is first released to cover the supra-arch branch vessels. The supra-arch branch vessels are then reconstructed by laser or mechanical reverse membrane rupture, followed by balloon dilatation and stent implantation. A study of 30 patients who underwent in situ fenestration for reconstruction of the left common carotid artery and brachiocephalic artery showed that the incidence of cerebral ischemia after in situ double fenestration was about 6.7% and the overall survival rate during the follow-up period was 92.3% [22]. Related studies have shown that the presence of double fenestrated stents has a profound impact on postoperative hemodynamics, causing stent migration or micro blood clots in the wall [23]. The long-term healing effect still needs to be further investigated.

For Z2 and some Z3 aortic lesions, stent graft needs to be implanted in Z1 to obtain a safe landing zone, and the left common carotid artery and left subclavian artery need to be reconstructed. The feasible methods include double fenestration, hybrid technique and chimney technique. There are few reports on the combination of Castor branched stent and fenestration technique [1]. Our study found that Castor combined with in situ fenestration achieves good clinical results in the treatment of aortic arch diseases, especially in the reconstruction of the superior 2nd arch branch. Our data showed that the single-branch Castor stent graft combined with in situ fenestration techniques of the left subclavian artery did not cause perioperative strokes, myocardial infarctions, cerebral infarctions, or other neurological complications, and no endoleak was observed. Therefore, based on the short-term clinical outcomes observed in our single-center retrospective cohort, we consider this method to demonstrate promising safety profiles that warrant further investigation.

While TEVAR is widely accepted as the standard treatment for thoracic aortic pathology, it remains associated with significant perioperative stroke risk—with published rates of 3% to 8% for TEVAR overall [24] and 0% to 11% specifically for TEVAR involving supra-arch branch reconstruction [25, 26], with a consistent trend: more complex arch reconstruction (i.e., more modules or vessels involved) correlates with higher cerebral complication rates [25–28]. The use of “Castor” in our study in combination with in situ fenestration treatment and the resulting clinical results resulted in similar satisfaction and encouraging findings regarding cerebral complications. In our study, the perioperative stroke rate was zero. Relevant studies have shown that the occurrence of stroke after TEVAR is related to perioperative hypotension, plaque embolism, and supra-arch branch coverage [29]. We hypothesize that stroke in fenestration-based aortic arch intervention may stem from left common carotid artery (LCCA) occlusion and intraprocedural stent fragment loss. In contrast, our Castor stent-graft use addressed these risks and avoided central nervous system (CNS) complications via its design and deployment: First, it directly reconstructed the LCCA, eliminating the need for additional procedures (e.g., in situ fenestration) that disrupt LCCA flow and raise occlusion risk. Second, deployment prioritized immediate LCCA perfusion: once the main stent was positioned, its single side branch was guided into the LCCA and instantly released post-main stent deployment. This restored LCCA blood flow promptly, bypassing in situ fenestration (linked to prolonged LCCA ischemia in traditional techniques). Additionally, the side branch anchored the main stent to the LCCA, preventing post-deployment migration.

Among the three aortic arch branches, the LSA presents the most challenging anatomical form for in-situ fenestration. Variations such as abnormal twisting, a small angle with the arch, narrow mounting, or the presence of atherosclerotic plaques can make it difficult for the sheath tip, inserted via the brachial artery, to maintain steady contact with the fabric portion of the stent graft [1]. We believe the following points should be noted when reconstructing the subclavian artery. Firstly, the characteristics of the arch should be clarified based on preoperative imaging data, and the surgical plan should be formulated accordingly. Before the operation, it is essential to confirm the involvement of the supra-arch branches and determine the number of reconstructions needed. For patients with severe vascular distortion of the target branch making reconstruction difficult, alternative plans must be developed. If an abnormal branch artery is found to be distorted or angled, strong radial support is needed to prevent postoperative bridge stent occlusion. Secondly, the distance between the aortic arch branches and the clock position of each supra-aortic branch should be measured. This allows for the optimal C-arm projection for the LSA with a tangential view to be determined preoperatively. This, combined with intraoperative DSA to find the best puncture position, can improve the success rate of the procedure. At the same time, this approach can prevent relative movement between the stent and the aortic wall caused by repeated punctures, reduce the loss of atherosclerotic plaque fragments, and minimize the risk of perioperative embolic stroke [29]. Although no MRI was performed to detect micro-infarctions, our data indicated that in situ laser fenestrations did not result in perioperative strokes, transient ischemic attacks, cerebral infarction, or other neurological complications, and no endoleak was observed. The laser fiber is flexible and can navigate various complex aortic arch anatomies, making it easier to penetrate PTFE grafts [18], such as those used in the Castor stent. This enhances the success rate of creating the fenestration.

The importance of thoracic false lumen thrombosis on aortic remodeling and long-term outcomes was confirmed by the Investigation of Stent Grafts in Aortic Dissection With Extended Length of Follow-up (INSTEAD-XL) trial [30]. The authors of this multicenter, randomized, prospective trial of TEVAR for uncomplicated dissection found that aorta-specific and all-cause mortality were reduced in patients randomized to TEVAR compared to those receiving optimal medical therapy alone. In our study, thoracic false lumen thrombosis occurred in 100% of patients treated with TEVAR, with no aorta-specific or all-cause mortality reported. However, there has been ongoing debate about abdominal false lumen thrombosis following TEVAR. Previous experimental studies have reported that distal entry tears exhibit bidirectional blood flow, which increases false lumen pressure and wall shear stress, potentially inhibiting the progression of false lumen thrombosis [31, 32]. Some researchers have suggested that achieving complete thrombosis of the false lumen may reduce the risk of late failure and prevent late rupture [33]. Zhu et al. described that distal entry tears in acute type B aortic dissection after thoracic endovascular aortic repair increase the occurrence of aortic events and inhibit aortic remodeling, but they do not significantly affect late survival [34]. Tang et al. investigated the causes of distal false lumen enlargement after type B dissection and concluded that Marfan syndrome, COPD, and the size of the primary tear were related [35]. At our center, the proximal entry tear is routinely treated, while distal entry tears are initially monitored during follow-up. In our cohort, no significant aortic expansion or compromised branch vessel perfusion was observed during follow-up. Intervention on distal tears is performed only if such changes develop. Prospective, large-scale, multicenter studies are needed to determine the optimal timing and strategies for managing distal entry tears. Although complete repair of all entry tears may benefit some patients, management should be individualized according to dissection anatomy to optimize long-term outcomes.

This study has several limitations. First, it is a retrospective analysis with a small sample size, and the follow-up period was relatively short. A larger sample size and a longer follow-up period are needed to better understand the long-term safety and efficacy of the Castor device. Second, although the results are presented as promising, there is no direct comparison with alternative endovascular methods such as double-branched stents or extracorporeal fenestration techniques. Third, long-term issues such as late stent migration, thrombosis, or endoleaks are only marginally addressed.

## Conclusions

This study demonstrates that the “Castor” single-branch stent combined with in situ fenestration left subclavian artery is a feasible, effective, repeatable, and safe strategy for reconstructing the branches of the aortic arch during TEVAR. The high technical success, low morbidity and mortality, and good early patency support its application in more patients with stable and safe procedures. However, longer follow-up is needed to assess efficacy and safety over time.

## Data Availability

Data can be acquired upon reasonable request to the corresponding.

## Abbreviations

TEVAR: Thoracic endovascular aortic repair
LCCA: Left common carotid artery
LSA: Left subclavian
CTA: Computed tomography angiography
CFA: Common femoral artery
LBA: Left brachial artery
SD: Standard deviation
IQR: Interquartile range
ISLF: In situ laser fenestration
D-SINE: Distal stent graft-induced new entry
P-SINE: Proximal stent graft-induced new entry
